# Supplementary material for: Volume Control for a Cortical Network
Source: bioRxiv. 2025 Jun 21:2025.06.19.660597. Preprint. [Version 1] doi: 10.1101/2025.06.19.660597 (PMC12262192; doi:10.1101/2025.06.19.660597)
Supplement: Supplementary file 1 [file NIHPP2025.06.19.660597v1-supplement-1.pdf]

bioRxiv preprint doi: https://doi.org/10.1101/2025.06.19.660597; this version posted June 21, 2025. The copyright holder has placed this preprint (which was not certified by peer review) in the Public Domain. It is no longer restricted by copyright. Anyone can legally share, reuse, remix, or adapt this material for any purpose without crediting the original authors.

Supplementary Material:

Control Experiment for Reference-Wire Artifacts in ESA Metrics

To confirm that reference-wire artifacts did not influence our ESA measurements, we performed control recordings in which anodic and cathodic iDC (±30 μA) were applied using identical stimulation and recording configurations in both living and euthanized rats. ESA relative percent changes during-vs.-before iDC were calculated for both conditions. Anodic and cathodic stimulation robustly altered ESA in layer 5 channels of the alive rat, whereas no change was observed across channels in the euthanized rat (**Supplemental Fig.1**). These results verify that ESA metrics reported here were unaffected by stimulation artifacts.

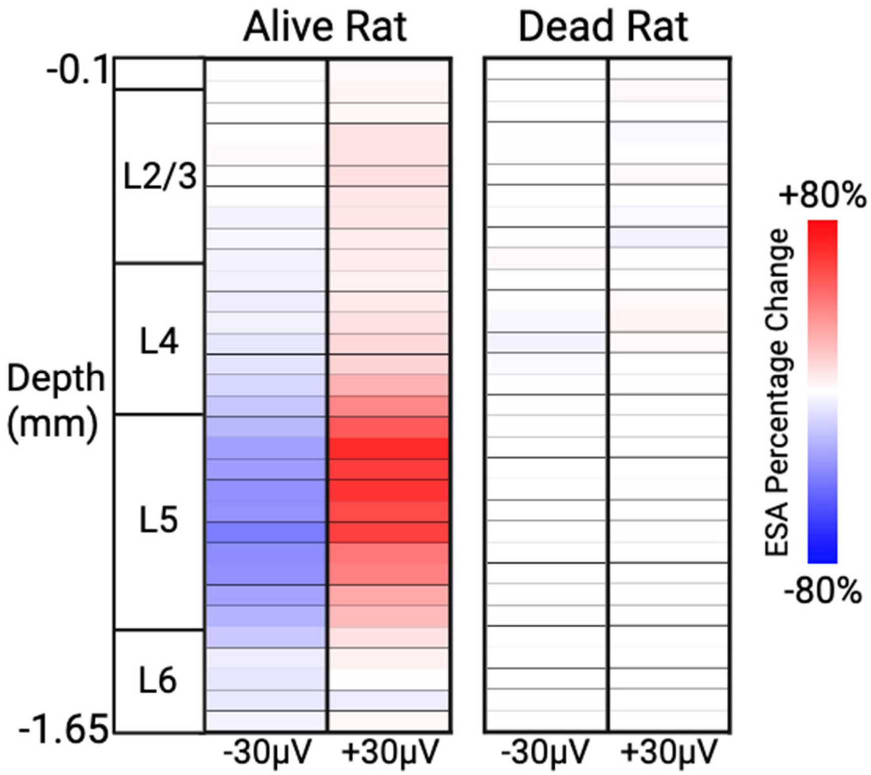

Supplemental Figure 1. Relative percent change in average ESA across recording channels for alive and euthanized rats.

bioRxiv preprint doi: https://doi.org/10.1101/2025.06.19.660597; this version posted June 21, 2025. The copyright holder has placed this preprint (which was not certified by peer review) in the Public Domain. It is no longer restricted by copyright. Anyone can legally share, reuse, remix, or adapt this material for any purpose without crediting the original authors.

Electric Field Mapping In Vivo

To validate the tissue resistivity parameter (ρ) used in our computational model, we delivered brief cathodic pulses (200 µs, -100 µA) through the iDC microcatheter placed at multiple lateral distances (0.25–2.6 mm) from the microelectrode array. Peak voltage amplitudes were recorded from all 32 channels spanning cortical depths (~0.10–1.65 mm) and lateral offsets (~0.25–2.55 mm), generating a two-dimensional voltage map of the induced electric field (**Supplemental Fig. S2**). We optimized the resistivity parameter by matching model-predicted voltages to experimentally recorded values, yielding a best-fit resistivity of approximately 5×10^3 Ω·mm, which was subsequently used in our computational model.

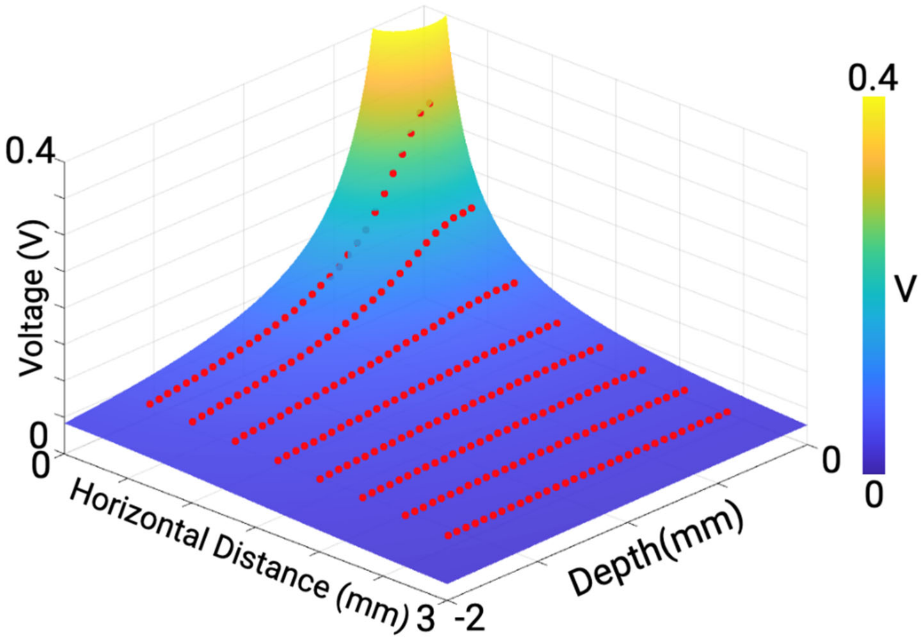

Supplemental Figure S2. Measured voltages (red dots) overlaid on model-predicted extracellular voltage spread.
